# Exosome Derived Micro RNA: A Promising Modality in the Treatment of Spinal Cord Injury and Peripheral Neuropathy

**DOI:** 10.34172/apb.46604

**Published:** 2026-05-10

**Authors:** Aminath Efa Ibrahim, Kannan Subbaram, Razana Faiz, Sheeza Ali, Queen Alice Arul

**Affiliations:** ^1^School of Medicine, The Maldives National University, Male’, Maldives; ^2^All India Institute of Medical Sciences, Kalyani, India

## To Editor,

 The spinal cord injury (SCI) and peripheral neuropathy (PN) are a spectrum of nervous system disorders that not only cause physical distress but also place a significant psychological burden on individuals with the diagnosis. The consequences of SCI and PN are both sudden and devastating, with high mortality and disability.

 SCI involves injury to the central nervous system. The initial insult that occurs is referred to as the primary injury, which is typically due to a mechanical force such as shear, laceration, or compression of the spinal cord. Within minutes of this, subsequent inflammatory processes are initiated, resulting in further injury, including ischemia, hypoxia, free radical production, and programmed cell death.^1^ This is the secondary injury, and it can take days to weeks to manifest. Peripheral neuropathy can arise from several pathologies such as trauma, infection, diabetic neuropathy, chemotherapy-induced neuropathy, and Guillain–Barré syndrome. The most common manifestation of PN is neuropathic pain, which can be debilitating.^1^

 Current management for spinal cord injury and peripheral neuropathy involves both medical and surgical measures. Medical management aims to reduce the extent of secondary injury following the primary insult. One such treatment modality is the use of steroids; the side effects that follow the treatment add to the burden and quality of life of individuals with the disease. Surgical techniques such as decompression increase blood flow; however, this only provides symptomatic relief.

 With advancing technology, targeted therapy involving microRNAs has become more prominent. MiRNA-based clinical therapies are primarily of two types: miRNA mimics, which reduce the expression of unfavourable genes, and miRNA inhibitors, which increase the expression of favourable genes. Dysregulation of miRNAs has been observed in the pathogenesis of SCI and PN. Hence, miRNA-based treatment focuses on reducing the inflammatory processes and promoting neurovascular recovery.

 One challenge noted, however, with using miRNA for therapeutic purposes was its short half-life in vivo. To overcome this, optimal delivery systems were studied. Among them, exosomal-based delivery systems were reported to have better outcomes.^2^ Exosomes are a subset of extracellular vesicles that play a role in intercellular communication. As these exosomes have a surrounding phospholipid layer, it protects exogenous miRNAs from hydrolysis by the extracellular enzymes. Hence, exosome-based miRNAs are more biocompatible compared to other polymeric-based carriers.^2^

 One example of such an exosomal delivery system is neuron-derived exosomal miR-124-3p. A preclinical study in which 45 male rats were treated with an intrathecal injection of 5 μL agomir-124 reported significantly greater improvement in functional outcomes compared to the control models at 21, 28, 35, and 42 days post-injury.^3^

 Studies on miR-21 and miR-9 revealed that they play a role in neuronal differentiation and axonal growth.^4,5^ Additionally, another study done in China reported that miRNAs combined with neuron-derived exosomes have a higher therapeutic efficacy.^6^

 Before transitioning from preclinical to clinical studies however, rigorous safety monitoring and standardized manufacturing processes needs to be established. The current exosome isolation methods rely on differential centrifugation or PEG precipitation, which yield 20-40% and 80-90% of exosomes, respectively. Although with PEG precipitation the yield is higher, the purity of the final product is significantly low.^7^ Additionally, because exosomes are prone to disruption at room temperature within minutes, the storage method must be uniform.

## Conclusion

 In conclusion, exosome-based miRNA therapy is a promising, non-invasive therapeutic approach that can significantly change how spinal cord and peripheral nerve injuries are managed. However, further research is necessary to establish the feasibility of this approach before it is made available to the community.

## Competing Interests

 The authors declare that there are no competing interests.

## Ethical Approval

 Not applicable.
